# Allergic sensitization does not influence advancement or survival in oral cancer

**DOI:** 10.1038/s41598-023-48879-8

**Published:** 2023-12-07

**Authors:** Lara Kakabas, Krzysztof Piersiala, Aeneas Kolev, Susanna Kumlien Georén, Lars-Olaf Cardell

**Affiliations:** 1 Division of ENT Diseases, Department of Clinical Sciences, Intervention and Technology, Karolinska Institutet, Stockholm, Sweden; 2 Department of Otorhinolaryngology, Karolinska University Hospital, Stockholm, Sweden; 3 Medical Unit Head Neck, Lung and Skin Cancer, Karolinska University Hospital, Stockholm, Sweden

**Keywords:** Oral cancer, Cancer, Adaptive immunity

## Abstract

A history of allergies has been said to be associated with a lower risk of head and neck cancer compared to the general population. However, it is not known whether having an allergic sensitization influences the prognosis and advancement of cancer disease. Thus, the aim of the study was to investigate the relationship between allergic sensitization and oral cancer advancement and patient survival. Allergen-specific IgE antibodies were investigated by ImmunoCAP™ Rapid in consecutive 80 patients with oral cancer. ImmunoCAP Rapid system tests a mix of representative inhalant allergens such as birch, timothy grass, mugwort, house dust mite, cat, dog, cockroach, olive (pollen), wall pellitory and mold. Eighty patients met the inclusion criteria for the study. Fifteen patients (19%) had positive ImmunoCAP test. There was no statistically significant difference in primary tumour size (T-stage) between groups (60% in allergy vs 68% in non-allergy had T1–T2 stage and 40% vs 32% T3–T4, respectively, p = 0.570). 27% of patients with allergy had nodal metastases compared with 37% of patients without allergy (p = 0.557). Both groups had comparable short-term survival. In conclusion, allergic sensitization does not seem to influence either the advancement or the short-term survival of patients with oral squamous cell carcinoma.

## Introduction

Head and neck cancer (HNC), is one of the leading cancers worldwide and a significant cause of morbidity and mortality. Risk factors include smoking, alcohol consumption and betel quid chewing. The most common histological type of HNC is squamous cell carcinoma (SCC). Oral squamous cell carcinoma is the most common type of HNC and approximately 380,000 new cases of oral cancer are diagnosed every year globally and numbers are predicted to increase^1^.

Numerous epidemiologic studies have shown a potential inverse association between allergies and some cancer types such as cancers of oesophagus, stomach, corpus uteri, lymphatic leukaemia and multiple myeloma^2–4^. On the other hand, some have found that the presence of allergies may actually increase the risk of multiple other types of cancer such as prostate^5^ and bladder cancer^4^, while odds ratios for lung^6,7^ and breast cancer^8^ are inconclusive. Several studies have also shown that a history of allergy is associated with a lower risk of HNC compared to the general population. A meta-analysis by Hsiao et al.^9^ based on 14 studies showed that a history of allergy is associated with a decreased risk of developing head and neck cancer with odds ratio (OR) of 0.8 (95% CI 0.6–0.9). One of the biggest studies based on two Finish registries^4^, where 78,000 asthmatic patients were included and analyzed, concluded that allergy protects against the onset of different tumours including oral and larynx cancer.

Despite these studies conducted on the risk of developing cancer, it is still unclear whether having allergy influences survival and disease advancement in oral cancer. Thus, the aim of the study was to investigate the relationship between allergic sensitization and oral cancer advancement and short-term survival of affected patients.

## Results

### Patients’ characteristics

Eighty patients met the inclusion criteria for the study. Clinicopathological characteristics of the two groups are presented in Table 1*.* Fifteen patients (19%) had allergic sensitization as defined in “Materials and methods”. The most common type of allergen was birch, which was observed in 73% of allergy patients. Other allergens detected were cat, dog, timothy, mold and house dust mite. The remaining 65 patients (81%) tested negative for presence of allergen specific IgE.

**Table 1:** Patient characteristics.

Characteristics	All cases N (%)	Allergy N (%)	Non-allergy N (%)	*p* value
Age mean (SD)	65.3 ± 14	60.9 ± 14	66.3 ± 14	0.188
Gender				
Male	47 (59)	6 (40)	40 (62)	0.128^a^
Female	33 (41)	9 (60)	25 (38)	
Smoking history				
Yes	45 (56)	6 (40)	39 (60)	0.160^a^
Never	35 (44)	9 (60)	26 (40)	
T staging (TNM)				
T1–T2	53 (66)	9 (60)	44 (68)	0.570^a^
T3–T4	27 (34)	6 (40)	21 (32)	
N staging (TNM)				
N0	52 (65)	11 (73)	41 (63)	0.557^b^
N+	28 (36)	4 (27)	24 (37)	
Grade				
1	8 (10)	1 (7)	7 (11)	0.247^b^
2	49 (61)	12 (80)	37 (57)	
3	23 (29)	2 (13)	21 (32)	
Tumor site				
Oral cavity				
Lip	1		1	
Uvula	1		1	
Tongue	53	10	43	
Gingiva	11	4	7	na
Floor of mouth	9	1	8	
Buccal mucosa	3		3	
Retromolar trigone	2		2	

^a^Pearson Chi-square test.

^b^Fisher.

There was no significant difference in smoking history between groups. Smokers constituted 60% of non-allergy group compared to 40% of the allergy group (p < 0.160). The most common type of cancer in the cohort was tongue cancer, which was observed in 66% of the allergy patients and in 67% of the non-allergy individuals.

### TNM staging in patients with allergy

To determine the influence of allergy on cancer advancement, we compared T- and N-stage of patients with and without allergy. There were no statistically significant differences in primary tumor size (T-stage) between groups (60% in allergy vs 68% in non-allergy had T1–T2 stage and 40% vs 32% T3–T4, respectively, p = 0.570). 27% of patients with allergy had nodal metastases (N+) compared with 37% of patients without allergy (p = 0.557). No significant difference was observed in tumor-grading; 7% in allergy vs 11% patients in non-allergy had grade 1. 80% vs 57% had grade 2 and 13% vs 32%, respectively, presented with stage 3.

### Overall survival (OS) in patients with allergy

**Figure 1:**
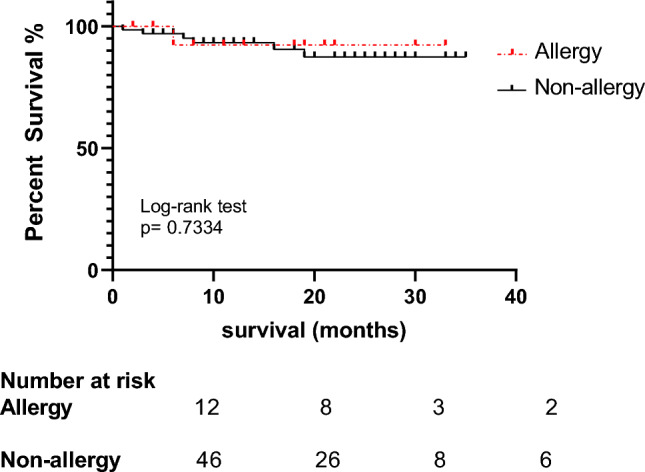
Kaplan–Meier curve comparing of overall survival between patients with and without allergy.

The 3-year OS rate was 92% in allergy patients while 87% rate was observed for non-allergy patients (Fig. 1). There was no statistically significant difference in survival between the two groups (Log-rank test, p = 0.7334).

### Use of antihistamines

The medical records of individuals who tested positive in the ImmunoCAP test were reviewed. It was found that among the 15 patients, 7 had a documented history of allergy in their medical records. Furthermore, 5 out of the 15 patients had been prescribed antihistamine medication and/or nasal steroids.

## Discussion

To our knowledge, this is the first study looking into correlation between allergic sensitization and survival in patients with oral squamous cell carcinoma. Based on our cohort, patients with allergy had comparable prognosis as patients without allergy. Furthermore, we showed that having allergy does not seem to influence the advancement (TNM staging) of the disease.

Allergy is a complex disorder and its influence on cancer disease is difficult to establish. Different types of allergies and even different allergens can potentially have a distant influence on the risk of cancer development and advancement of the disease. In this project, we investigated patients with allergy to most-common airborne allergens in Swedish population such as: birch, timothy grass, mugwort, house dust mite (D. pteronyssinus), cat, dog, cockroach, olive (pollen), wall pellitory and mold (A.alternata). The prevalent symptoms of airborne allergies include sneezing, coughing, red and itchy eyes, swelling, or wheezing. Lindelöf et al.^10^ looked previously into relationship between the risk of developing cancer and positive Phadiotop test in Swedish population. They did not find any correlation between having allergies and risk of cancer. Our study results extend their finding by showing that having allergy does not have a major impact on advancement of cancer and survival of patients with oral cancer. In our cohort, there was no statistically significant difference in size of the tumour or nodal advancement between patients with and without allergy. To our knowledge, this is the first report looking into this dependence in patients with oral squamous cell carcinoma.

In recently published studies, the main two theories have been discussed and proposed to justify the inverse association between allergies and cancer. According to the “immunosurveillance hypothesis”, allergy is involved in the immune system’s ability to recognize and eliminate tumour cells^11^. The second theory, the “prophylaxis hypothesis”, suggests that allergy symptoms are the body’s way of expelling potential carcinogens and pathogens. In this way, allergy plays a vital role in lowering the risk of cancer^11,12^. Previous studies suggest that IgE may have a natural immunological surveillance function in some malignancies^13^. Furthermore, IgE antibodies were shown to have a direct cytotoxic influence on malignant cells^11,14^. Ferastraoaru et al. in a recent review^15^ emphasized that low or absent IgE levels may impede anti-tumor surveillance, as a correlation was observed between low IgE levels and a significantly higher risk of malignancy development. The role of IgE in anti-tumor immunity begins to be recognized; however, further studies are required to comprehensively understand its functions.

Several aforementioned studies showed lower risk of certain cancer types, including HNC, in populations with allergies compared to general population. A meta-analysis by Hsiao et al. indicated a statistically significant inverse association between head and neck cancer and allergies^9^. In particular, the association was clear for allergic rhinitis and was more pronounced among men. The inverse association favors the immune surveillance theory. However, no studies looking into the influence of allergy on prognosis or advancement of head and neck cancer have been performed.

A limitation of this study is that it is restricted to one center with a limited number of enrolled subjects. Furthermore, the follow-up time is short and does not allow analysis of the 5-year survival. Moreover, the methods employed did not include the measurement of specific IgE levels. Although IgE levels were not measured, we believe that the presence or absence of allergic sensitization can profoundly impact the immune system. Still, this is the first prospective study investigating the presence of IgE antibodies at the time of surgery in cancer patients. We plan also to follow up our patients to investigate the influence of allergy on long-term survival. Other potential confounding factors, which have not been analysed within the scope of this project, are the use of anti-allergy medication and total IgE level in the blood of enrolled patients.

In conclusion, based on our cohort, allergic sensitization does not seem to influence either the advancement or the survival of patients with oral squamous cell carcinoma.

## Materials and methods

### Subjects

Eighty consecutive patients with oral squamous cell carcinoma who were willing to participate in the study and provided a written informed consent were enrolled. The age range was 23–91. Mean age equaled 65 years. There were 47 male patients (59%) and 33 female patients (31%). All patients underwent surgical excision of the primary tumour and sentinel node biopsy at Karolinska University Hospital, Stockholm, Sweden between December 2019 and September 2022. Patients provided two blood samples on the day of the surgery which were further analysed at the laboratory as described under. Eligible patients enrolled for this study met following inclusion criteria: (1) diagnosis of primary oral cancer squamous cell carcinoma (OSCC) (2) tumour excision with sentinel node biopsy, (3) willingness to participate in the study. Exclusion criteria were as follows: (1) systematic autoimmune diseases (2) synchronous second malignancies, hemo-lymphopoietic malignancies in the past (3) any other acute or chronic condition that could influence immunological system.

### Allergic sensitization diagnosis

Allergen specific IgEs were detected by ImmunoCAP™ Rapid (Thermo Fisher Scientific, Uppsala, Sweden), according to the manufacturer’s instructions. The ImmunoCAP is a miniaturized immunoassay platform (multiple allergen components immobilized on a slide) that is used to assess the presence of multiple antibodies in a single blood test. ImmunoCAP Rapid is a lateral flow test which means that the whole blood sample is applied to the sample well and the separated plasma portion flows onto the test strips. IgE antibodies present in the sample bind to the relevant area on the strip representing an allergen. The developer solution is then applied and it releases the dried gold-anti-IgE conjugate. The conjugate forms a complex with the already bound IgE antibodies. When the reaction takes place, there is a visible pink-red line in the Test window, representing positive test result. ImmunoCAP Rapid system tests a mix of representative inhalant allergens such as birch, timothy grass, mugwort, house dust mite (D. pteronyssinus), cat, dog, cockroach, olive (pollen), wall pellitory and mold (A. alternata).

### Statistical analysis

Statistical analyses were performed with GraphPad Prism version 9.0.0 (GraphPad Software, La Jolla, CA, USA). The Fisher’s exact test (if n < 5) or Chi square test was used to test the dependence between clinicopathological descriptive features and presence of IgE against selected airborne allergens. Survival analysis was performed by log-rank test comparison. p-value < 0.05 was considered significant.

### Ethical approval

All procedures performed in studies involving human participants were in accordance with the ethical standards of the institutional and/or national research committee and with the 1964 Helsinki declaration and its later amendments or comparable ethical standards. Informed consent was obtained from all individual participants included in the study. Ethics Committee Approvals: 2015/1650-31/2, 2019-03518 and 2021-01265.

## Data Availability

The authors confirm that the data supporting the findings of this study are available within the article.
